# The Effects of Vitamins and Micronutrients on *Helicobacter pylori* Pathogenicity, Survival, and Eradication: A Crosstalk between Micronutrients and Immune System

**DOI:** 10.1155/2022/4713684

**Published:** 2022-03-16

**Authors:** Ali Nabavi-Rad, Mahsa Azizi, Shaghayegh Jamshidizadeh, Amir Sadeghi, Hamid Asadzadeh Aghdaei, Abbas Yadegar, Mohammad Reza Zali

**Affiliations:** ^1^Foodborne and Waterborne Diseases Research Center, Research Institute for Gastroenterology and Liver Diseases, Shahid Beheshti University of Medical Sciences, Tehran, Iran; ^2^Gastroenterology and Liver Diseases Research Center, Research Institute for Gastroenterology and Liver Diseases, Shahid Beheshti University of Medical Sciences, Tehran, Iran; ^3^Basic and Molecular Epidemiology of Gastrointestinal Disorders Research Center, Research Institute for Gastroenterology and Liver Diseases, Shahid Beheshti University of Medical Sciences, Tehran, Iran

## Abstract

*Helicobacter pylori* as a class I carcinogen is correlated with a variety of severe gastroduodenal diseases; therefore, *H. pylori* eradication has become a priority to prevent gastric carcinogenesis. However, due to the emergence and spread of multidrug and single drug resistance mechanisms in *H. pylori*, as well as serious side effects of currently used antibiotic interventions, achieving successful *H. pylori* eradication has become exceedingly difficult. Recent studies expressed the intention of seeking novel strategies to improve *H. pylori* management and reduce the risk of *H. pylori*-associated intestinal and extragastrointestinal disorders. For which, vitamin supplementation has been demonstrated in many studies to have a tight interaction with *H. pylori* infection, either directly through the regulation of the host inflammatory pathways or indirectly by promoting the host immune response. On the other hand, *H. pylori* infection is reported to result in micronutrient malabsorption or deficiency. Furthermore, serum levels of particular micronutrients, especially vitamin D, are inversely correlated to the risk of *H. pylori* infection and eradication failure. Accordingly, vitamin supplementation might increase the efficiency of *H. pylori* eradication and reduce the risk of drug-related adverse effects. Therefore, this review aims at highlighting the regulatory role of micronutrients in *H. pylori*-induced host immune response and their potential capacity, as intrinsic antioxidants, for reducing oxidative stress and inflammation. We also discuss the uncovered mechanisms underlying the molecular and serological interactions between micronutrients and *H. pylori* infection to present a perspective for innovative in vitro investigations, as well as novel clinical implications.

## 1. Introduction

As a Gram-negative pathogen, *Helicobacter pylori* (*H. pylori*) is uniquely adapted to survive in the acidic environment of the human stomach [1]. *H. pylori* infection most frequently acquires in childhood and remains generally asymptomatic with long-term clinical sequelae. The incidence of the infection is over half of the world's population and is highly dependent on geographical origin and socioeconomic status [2].

*H. pylori* infection leads to gastric mucosal damage by means of its virulence factors, induction of host proinflammatory cytokines, reactive oxygen species (ROS), and reactive nitrogen species [3]. The mucosal damage can progress to gastritis, peptic ulcer disease (PUD), dyspepsia, gastric mucosa-associated lymphoid tissue (MALT) lymphoma, or gastric carcinoma [4]. Considering *H. pylori* as the major cause of gastric carcinoma, the World Health Organization (WHO) lists *H. pylori* as a class I carcinogen [5]. Despite the reduction in infection prevalence owing to antibiotic usage, the eradication of *H. pylori* has become an important matter to prevent gastric cancer [6].

However, it has been well established, achieving successful eradication of *H. pylori* infection is exceedingly difficult [7]. As the first-line of treatment, international guidelines suggest four drug combinations with two or three types of antibiotics for 10-14 days [8]. Even the simplest regimen, proton-pump inhibitor (PPI) and amoxicillin may come up with serious adverse effects including type 2 diabetes and increase the risk of gastric adenocarcinoma [9–11]. As a result, currently used treatments are not sufficiently effective, so alternative therapies need to be developed [12].

Even though there are contrary opinions on the effect of vitamin administration, several studies demonstrated that *H. pylori* infection decreases certain vitamin concentration in the gastric juice [13]. Many studies even showed a substantial increase in the eradication rate by vitamin supplementation in a dose-dependent manner [14]. On the other hand, various minerals are reported to directly or indirectly regulate *H. pylori* colonization and pathogenicity [13]. In this review, we discuss the current knowledge on how vitamins and minerals may link to *H. pylori* eradication or the host immune defenses against this recalcitrant pathogen. We also highlight the latest findings regarding vitamin and mineral interventions affecting the pathogenesis of *H. pylori* to elucidate the importance of micronutrients supplementation, providing a hopeful perspective for the development of potential preventive and therapeutic applications in clinical practice.

## 2. *H. pylori* and Immune System

The gastrointestinal mucosa, frequently encountered with antigens and food allergens, is equipped by a mucosal immune system to protect it against harmful foreign materials [15]. The gastrointestinal mucous layer and epithelial layer form the mucosal surface which is supported by the lamina propria underneath the epithelium [16]. Gastrointestinal immune components can be classified into either inductive sites including the mesenteric lymph nodes (MLN) and the gut-associated lymphoid tissues (GALT) or effector sites including the gut lamina propria and epithelial layer, in which they undergo maturation or localization, respectively [17]. There are antibodies, cytokines, chemokines, and their receptors in gut mucosal effector sites to provide immune protection and maintain homeostasis [18].

*H. pylori* is correlated with chronic inflammation, causing structural changes in gastric mucosa including glandular atrophy and intestinal metaplasia (IM) and developing gastric carcinoma [19]. The main explanation of inflammatory changes stimulated by this pathogen is the enhanced production of interleukin-1*β* (IL-1*β*), IL-8, IL-12, tumor necrosis factor-*α* (TNF-*α*), and ROS [20]. As a result of *H. pylori* infection, neutrophils, eosinophils, mast cells, and dendritic cells (DCs) infiltrate to the mucosal epithelium to provoke active inflammation [21]. Moreover, by the attraction and activation of neutrophils, *H. pylori* enhances the production of TNF-*α*, IL-1*β*, IL-8, ROS, macrophage inflammatory protein-1*α* (MIP-1*α*), and MIP-1*β* [22]. IL-1*β* increases the adhesion of neutrophils to endothelial cells by stimulating the expression of adhesion molecules. Both TNF-*α* and IL-1*β* enhance the secretion of prostacyclin (PGI2), prostaglandin E2 (PGE2), and platelet-activating factor (PAF) from endothelial cells [23]. As lipid mediators, PGE2 and PGI2 have a role in the regulation of vascular function and inflammation. In particular, PGE2 regulates the permeability of endothelial cells, and PGI2 exhibits a protective impact during the resolution phase of inflammation [24]. The PAF signaling cascade activates a variety of intracellular pathways in inflammatory and immune cells and changes cellular function and phenotype to trigger or expand inflammatory response [25].

IL-8, a chemokine that plays a critical role in *H. pylori*-induced inflammation, activates the CD11b/CD18 dimer to form a complex with neutrophils. This complex binds to ICAM-1 on the membrane of vascular endothelial cells and forms a tetramer; therefore, IL-8 recruits neutrophils and promotes their adhesion to endothelial cells [26]. As a result, excessive production of IL-8 leads to neutrophil influx and inflammation [27]. On the other hand, ROS including superoxide anion (O_2_^−^), hydroxyl radical (OH•), hydrogen peroxide (H_2_O_2_), and singlet oxygen (^1^O_2_), are natural by-products of normal cell activity, mainly generated in the respiratory chain in mitochondria and photochemical and enzymatic reactions [28]. ROS, in their normal concentration, act as the intermediate in cell signaling pathways. On the contrary, the overproduction of ROS is associated with various diseases, inflammation, DNA damage, and interfering cell signaling pathways. The homeostasis of ROS is a major priority for living organisms to prevent genome instability and cell death [29].

Depending on the bacterial virulence genotype, *H. pylori* infection results in excessive production of ROS and developing oxidative stress [30]. *H. pylori* bacteria, especially cytotoxin-associated gene A (CagA)-positive strains, induce the activation of the enzyme mitogen oxidase-1 (Mox1) in epithelial cells, leading to enhanced generation of superoxide anions [31]. It is long known that releasing various inflammatory mediators, such as chemokines which are chemoattractants of neutrophils from *H. pylori*, stimulates neutrophils to produce ROS in a normal concentration [32]. Because one of the major immune responses of neutrophils is the generation of oxidative bursts by NADPH oxidase to kill the invasive pathogen, persistent inflammatory mediators increase the risk of oxidative stress [33]. Moreover, *H. pylori* neutrophil-activating protein (HP-NAP), released from *H. pylori* possibly by autolysis, induces the overexpression of ROS and chemokines [22].

Macrophage chemotactic protein-1 (MCP-1) is released from epithelial cells to induce the recruitment of macrophages and consequently release IL-1*β* as an inflammatory response. *H. pylori* infection promotes the expression of MCP-1. Accordingly, macrophage activation and excessive IL-1*β* secretion lead to gastric inflammation [34]. Activated macrophages also develop oxidative stress by enhanced ROS production, mainly through NADPH oxidase-2, xanthine oxidase, and the mitochondrial electron transport chain [35]. It is suggested that *H. pylori* lipopolysaccharide (LPS) might activate the macrophages. However, the meticulous effect of *H. pylori* LPS on macrophage activation is yet to be elucidated [36, 37].

In gastric epithelial cells, vascular endothelial cells or immune cells, especially macrophages, *H. pylori* promotes the production of inducible nitric oxide synthase (iNOS) which stimulates nitric oxide (NO) production. As a highly reactive compound, NO frequently reacts with O_2_^−^ to generate highly toxic peroxynitrite (ONOO^−^) [38]. However, *H. pylori* can inhibit the bactericidal activity of ONOO^−^ by converting it to nitrosoperoxycarbonate (ONOOCO_2_^−^) with urease product CO_2_; therefore, it causes collateral damage to host cells and does not eliminate *H. pylori* [39]. On the other hand, *H. pylori* is equipped with catalase and superoxide dismutase to detoxify ROS and also able to downregulate nitric oxide generation by means of arginase enzyme [40].

The innate immune system as the first-line of defense is equipped with recognition mechanisms, allowing detection and response to antigens without prior recognition [41]. *H. pylori* contains pathogen-associated molecular patterns (PAMPs) such as peptidoglycan, LPS, lipoproteins, and flagellins which are recognized by pattern recognition receptors (PRRs) such as Toll-like receptors (TLRs), C-type lectin receptors (CLRs), NOD-like receptors (NLRs), and RIG-like receptors (RLRs) [42]. PAMP detection leads to multiple signaling pathways that increase nuclear factor kappa B (NF-*κ*B) and activated protein-1 (AP-1) activation and immune effector's expression [43]. NF-*κ*B and AP-1 are transcriptional factors, regulating a variety of gene expressions that contribute to important cellular activity, such as cell proliferation, apoptosis, and the innate and adaptive immune responses [44, 45]. To bind DNA and regulate gene transcription, NF-*κ*B and AP-1 must be activated and translocated from the cytoplasm to the nucleus. The major activators for NF-*κ*B and AP-1 by *H. pylori* are CagA and LPS [46, 47]. Adhesion molecules of *H. pylori*, antigen binding adhesion A (BabA), sialic acid binding adherence A (SabA), or outer membrane protein Q (HopQ), allow attachment of the bacteria to gastric epithelial cells [48]. LPS binds to TLR4, a member of signal transduction proteins, to induce the expression of transcriptional factors including NF-*κ*B and AP-1 mostly in macrophages and gastric epithelial cells [49]. On the other hand, CagA needs to be injected into the host cell by type IV secretion system (T4SS) machinery to interact with intramembrane hepatocyte growth factor (HGF) receptor Met or epidermal growth factor receptor (EGFR) for upregulation of NF-*κ*B and AP-1 [50, 51]. The activation of NF-*κ*B and AP-1 stimulates the transcription of inflammatory mediators such as ROS and cyclooxygenase-2 (COX-2), inflammatory cytokines such as IL-8 and IL-6, and tumor necrosis factor receptor-associated factor (TRAF) genes, resulting in several cellular proliferation and damages [52, 53].

To survive the low pH (1.5 to 3.5) [54] environment of the stomach, *H. pylori* catalyzes the hydrolysis of urea by urease to produce ammonia (NH_3_), thereby buffering the cytoplasm, periplasm, and immediate environment surrounding the bacteria. The urease is comprised of two major subunits UreA and UreB, encoded by the urease operon, accessory proteins for maturation, and two pivotal Ni^2+^ ions for activation [55]. Independent to enzymatic activity, urease binds to the CD74 receptor on gastric epithelium cells to activate inflammatory pathways, leading to enhanced IL-8, IL-4, and T helper (Th) 1 cytokine secretion, NF-*κ*B and macrophage activation, epithelial tight junction interference, and platelet aggregation [56].

*H. pylori* strains, possessing virulence factors such as CagA and vacuolization cytotoxin A (VacA), elevate the risk of atrophic gastritis and gastric cancer. Using a T4SS apparatus which is encoded by *cag* pathogenicity island (*cag*PAI), *H. pylori* injects CagA oncoprotein into host cells and mainly activates NOD1 [57]. Consequently, CagA increases the activation of transcription factors such as NF-*κ*B, AP-1, and mitogen-activated protein kinase (MAPK) and IL-8 expression [58]. Moreover, CagA possibly permits VacA to break into the gastric submucosa and connect with macrophages, DCs, and B and T cells by opening the tight junctions [59]. VacA can prevent T cell activation and proliferation by downregulation of nuclear factor of activated T cells (NFAT) and therefore blocking the secretion of IL-2 [60].

## 3. *H. pylori* and Vitamins

### 3.1. Vitamin D

Vitamin D, a fat-soluble vitamin, has few dietary sources, and thereby, the primary route of obtaining this vitamin is through dermal synthesis after ultraviolet-B (UVB) radiation [61]. The two primary forms of vitamin D are vitamin D2 and vitamin D3, formed from ergosterol and 7-dehydrocholesterol by UVB radiation, respectively [62]. Vitamins D2 and D3 are prohormones and biologically inactive. They need a 2-step enzymatic hydroxylation process to be activated [63]. The first step is performed by 25-hydroxylase in the liver, converting the components to 25 (OH) D (calcidiol). Then, the 25 (OH) D undergoes 1*α*-hydroxylation mainly in the kidney to form 1, 25 (OH)2 D (calcitriol), the most active form of vitamin D [64]. It has been demonstrated that cytokines such as interferon-gamma (IFN-*γ*) stimulate the production of 1, 25 (OH)2 D by monocytes [65].

The genomic actions of vitamin D, such as downregulation of IL-2 and IFN-*γ* expression by inhibition of transcription factors, are through vitamin D receptor (VDR), a member of nuclear hormone receptor family, which will further bind to specific DNA regions named vitamin D response elements (VDRE) [66]. The nongenomic activities of vitamin D include calcium homeostasis and regulation of hormone secretion, immune function, and cellular proliferation [67]. Although the whole molecular mechanisms of vitamin D activity are yet to be discovered, it has been suggested that vitamin D regulates both transcellular and paracellular calcium absorptions in the intestine [68]. Vitamin D induces calcium uptake across the brush border through calcium selective channel TRPV6. It also increases the production of calbindin, the calcium-binding protein, to stimulate calcium transport through the cell. Vitamin D stimulates the generation of calcium pump as transcellular actions and downregulates cadherin 17 and aquaporin 8 as paracellular activities [69]. In the case of calcium homeostasis, vitamin D also regulates the calcium reabsorption in the distal tubule of the kidney [70]. Vitamin D downregulates parathyroid hormone secretion but stimulates insulin secretion, downregulates cellular proliferation but stimulates cellular differentiation such as the osteoblast by regulating the synthesis of collagen and alkaline phosphatase proteins [71, 72].

### 3.2. Vitamin D and Immune Function

Micronutrients including vitamin A, D, C, E, B6, B9, B12, zinc, iron, copper, nickel, and selenium play a key role in immune responses [73]. Cathelicidin antimicrobial peptide (CAMP) and *β*-defensin, mainly expressed and stored in epithelial cells and macrophages, demonstrate antibacterial actions against Gram-negative and Gram-positive bacteria and fungi [74, 75]. Calcitriol boosts the effect of antimicrobial proteins of macrophages and monocytes such as CAMP and *β*-defensin, regulating gut microbiota composition and preserving the normal bacterial community, therefore supporting the integrity of the gut barrier [76, 77]. On the other hand, it enhances the differentiation of monocytes to macrophages and boosts the movement and phagocytic ability of macrophages [78]. By increasing the expression of anti-inflammatory cytokines, calcitriol downregulates the production of IFN-*γ* and proinflammatory cytokines [79]. Tight junction CLDN2 gene is a direct target of VDR; therefore, vitamin D increases the expression of tight junction protein that plays a major role in intestinal barrier function and homeostasis [80]. It has been also reported that vitamin D plays a pivotal role in preserving renal epithelial barrier function [81].

### 3.3. The Effect of Vitamin D on *H. pylori* Infection and Eradication

**Figure 1:**
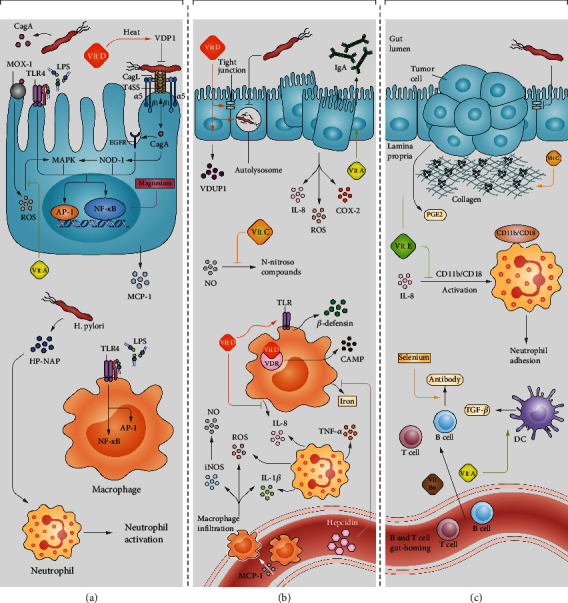
The effect of micronutrients on *H. pylori* pathogenesis. (a) *H. pylori* can invade epithelial cells and interact with epidermal growth factor receptor (EGFR), toll-like receptor 4 (TLR4), and mitogen oxidase-1 (Mox1), thereby stimulating the activation of transcriptional factors and the production of reactive oxygen species (ROS). *H. pylori* neutrophil-activating protein (HP-NAP) and LPS lead to neutrophil activation and promotion of macrophage inflammatory responses, respectively. However, vitamin D and A interfere with CagA expression and ROS-mediated mitogen-activated protein kinase (MAPK) activation, respectively. Furthermore, vitamin D3 decomposition product (VDP1) lyses the bacterial cell and inhibits the colonization of *H. pylori*. Magnesium downregulates the activation of nuclear factor kappa B (NF-*κ*B) and activated protein-1 (AP-1); therefore, suppressing the host inflammatory responses. (b) Neutrophil activation promotes the production of tumor necrosis factor-*α* (TNF-*α*), interleukin 8 (IL-8), IL-1*β*, and ROS. Macrophage chemotactic protein-1 (MCP-1) stimulates the infiltration of macrophages and thereby increases the concentration of inducible nitric oxide synthase (iNOS), IL-1*β*, and ROS in the gut lamina propria. The infected epithelial cells overexpress proinflammatory cytokines such as cyclooxygenase-2 (COX-2), IL-8, and ROS. The overproduction of hepcidin in the liver, owing to *H. pylori* infection, interferes with the iron release from the macrophages. Vitamin D and A promote the expression of Vitamin D upregulated protein 1 (VDUP1) and immunoglobulin A (IgA), respectively. Regarding macrophages, vitamin D inhibits IL-8 secretion, increases *β*-defensin production through TLR activation and provokes the production of cathelicidin antimicrobial peptide (CAMP) by vitamin D receptor (VDR). Vitamin D regulates the reduced function of autolysosome and consequently stimulates *H. pylori* degradation in gastric epithelial cells. Moreover, this vitamin promotes the gut epithelial barrier by increasing the expression of tight junctions. On the other hand, vitamin C can reduce N-nitroso compound formation from nitric oxide (NO) and thereby inhibits carcinogenesis. (c) As chronic inflammation results in carcinogenesis, vitamin C promotes collagen production and prevents tumor metastasis. On the other hand, vitamin E reduces neutrophil adhesion and the secretion of prostaglandin E2 (PGE2) from tumor cells. Vitamin A and B6 induce B and T cell gut-homing, and selenium provokes the antibody secretion from B cells. Vitamin A is also involved in the production of transforming growth factor-beta (TGF-*β*) in the dendritic cells (DCs).

Vitamin D was found to suppress the *H. pylori*-induced production of proinflammatory cytokines including IL-1, IL-2, IL-6, IL-8, and TNF-*α* (Figure 1) [82]. Dauletbaev et al. similarly reported that vitamin D administration in the cell culture system suppresses the production of IL-8 in hyperinflammatory macrophages independent of the DUSP1 gene (an anti-inflammatory gene that partly regulates IL-8 production) [83]. A recent study demonstrated that vitamin D supplementation leads to downregulation of IL-8 in *H. pylori*-infected mice, in both wild type and VDR knockdown [84].

Antimicrobial peptides (AMPs) have a variety of inhibitory effects against pathogens and play a major part in the innate immune system of many organisms [85]. Although the exact mechanisms are yet to be discovered, Merriman et al. exhibited that vitamin D enhances the expression of multiple genes of *β*-defensin in vivo [86]. In humans, IL-1*β* and TLR activation are required for vitamin D to upregulate *β*-defensin [87]. Vitamin D also increases the expression of VDR which positively upregulates the production of vitamin D in the body; moreover, the vitamin D/VDR complex will further bind to CAMP promotor and significantly enhance its expression [88, 89]. Guo et al. indicated that the presence of small interfering (si) VDR and siCAMP in the individuals might reject the theory that vitamin D has antibacterial activity [89]. However, by regulating the expression of CAMP and *β*-defensin, vitamin D has a crucial role in the immune response against various pathogens including *H. pylori* [90].

As a transmembrane glycoprotein, EGFR is involved in various cellular activities including proliferation, differentiation, and apoptosis which highlights the significance of this transmembrane receptor in mucosal defense. On the other hand, EGFR activation is of great significance to inflammatory and repair processes such as MAPK phosphorylation [91]. Consequently, EGFR not only mediates *H. pylori*-induced inflammation but also plays a crucial role in lung cancer and it has been discovered that vitamin D supplementation comes up with a possible advantage in the treatment. As a result, it is a possibility that vitamin D inhibits EGFR in *H. pylori*-infected individuals and suppresses the inflammation [92, 93]. In a dose-dependent manner, it also appears that vitamin D supplementation significantly reduces the CagA expression and suppresses *H. pylori*-induced production of IL-6 and IL-8 in animal models. Subsequently, vitamin D might be able to suppress *H. pylori*-induced inflammation [84].

Autophagosome fusion with lysosomes forms autophagolysosomes, which eliminates pathogens after their detection by the innate immune system [94]. *H. pylori* inhibits the lysosomal function by decreasing the lysosomal clearance of infected autophagosomes, resulting in bacterial survival [95]. Hu et al. demonstrated that vitamin D restores the impaired lysosomal activity of epithelial cells through stimulating membrane receptor PDIA3, leading to the redistribution of PDIA3-STAT3 complex into the nucleus and thereby enhancing the production of MCOLN3 protein, both in vitro and in vivo [96]. MCOLN3 channel is responsible for Ca^2+^ trafficking which has a pivotal role in lysosomal acidification [97]. Consequently, downregulated expression of MCOLN3 protein by *H. pylori* can be normalized by vitamin D treatment, leading to the elimination of *H. pylori* [96].

VDP1, vitamin D3 decomposition product, destabilizes the membrane structure of *H. pylori* and lyses the bacterial cell independent of blocking the cellular metabolism through binding to dimyristoyl phosphatidylethanolamine (DMPE) in membrane lipid composition of *H. pylori* [98]. The anti-*H. pylori* effect of VDP1 is extremely selective and commonplace bacteria can survive even in a high concentration of VDP1 [99].

Vitamin D upregulated protein 1 (VDUP1), playing several roles in a variety of cellular processes such as the suppression of TNF-*α*-induced NF-*κ*B activation, is upregulated by vitamin D supplementation [100, 101]. In animal models, VDUP1 reduces *H. pylori*-induced gastric carcinogenesis by regulating the maturation and cytotoxicity of natural killer (NK) cells and downregulating the expression of TNF-*α*, NF-*κ*B, and COX-2 [102, 103].

Regarding the *H. pylori* infection in infants and toddlers, Gao et al. demonstrated that the prevalence of vitamin D deficiency in children with *H. pylori*-positive antibody was more likely to occur than the *H. pylori*-negative antibody group [104]. Concerning adults, Shafrir et al. demonstrated that vitamin D was inversely related to *H. pylori* infection and the possibility of infection was significantly higher in vitamin D-deficient patients than vitamin D-sufficient patients [105]. A recent study similarly reported that in the old patients, aged 65 years, with sarcopenia, *H. pylori* infection led to vitamin D deficiency and the prevalence of infection was significantly higher in vitamin D-deficient patients [106]. Twenty years of 1*α*-hydroxyvitamin D3 supplementation resulted in a significantly lower *H. pylori* infection rate in subjects [107]. In the case of *H. pylori* eradication, among 150 *H. pylori*-infected patients, Shahawy et al. reported that the mean level of 25 (OH) D was substantially higher in the successful eradication group [108]. Having a serum vitamin D level < 10 ng/mL is possibly an independent risk factor for the treatment failure of *H. pylori* infection [109].

Although there are controversial results regarding the efficiency of vitamin D supplementation during *H. pylori* eradication, multiple cohort studies and clinical trials mostly reported a reverse interaction between serum vitamin D level and *H. pylori* infection as it is supported by several in vitro antipathogenic activities of this vitamin (Table 1). Furthermore, vitamin D administration might reduce the adverse effects of antibiotic treatment by preserving the normal gut microbiota composition.

### 3.4. Vitamin A

Vitamin A, a group of fat-soluble vitamins, was discovered in 1906, and owing to the crystallization of vitamin A in 1937, it was synthesized for the first time by Otto Isler in 1947 [111]. This vitamin is essential in preserving physiological activities such as vision, growth, and integrity of epithelial and mucosal tissues as well as regulating the host immune responses [112]. The most common forms of food-derived vitamin A are retinol (vitamin A1) and carotenoids (provitamin A) which are transferred to hepatic stellate cells (HSCs) and then stored as retinyl ester [113]. Retinol, especially found in animal foods including dairy, fish, egg yolks, and meat, is converted to retinal by retinyl ester and thereby plays a key role in low-light and color vision and mitochondrial oxidative phosphorylation [114, 115]. By being converted to retinoic acid (RA), a hormone-like growth factor, retinol stimulates the differentiation and growth of epithelial cells and maintains the homeostasis of the skin and bone [116, 117].

Carotenoids or provitamin A are a complex collection of more than 850 naturally existing pigments, which are produced by plants, algae, and photosynthetic bacteria [118]. As the human body is incapable of carotenoid synthesis, its bioavailability highly depends on dietary resources [119]. *β*-carotene is the most common provitamin A [120] and its bioavailability is affected by several factors, such as food processing, nutritional matrix composition, and gastrointestinal health status [121]. Although *β*-carotene can scavenge free radicals, it is incapable of being regenerated after decomposition; therefore, it is suggested that the main bioactivity of *β*-carotene is that of a provitamin A [122].

### 3.5. Vitamin A and Immune Function

The active metabolite of vitamin A plays a major role in epithelial tissue differentiation and mucosal immune responses [123]. RA induces gut-homing specificity in B and T cells by increasing the expression of chemokine receptor CCR9 on these cells, thereby promoting their trafficking to intestinal tissue [124]. Vitamin A also affects T cell regulation and differentiation into different T helpers, including Th1, Th2, and Th17 cells [125]. It has been discovered that RA enhances the secretion of transforming growth factor-beta (TGF-*β*) from DCs, therefore regulating the adaptive immune response [126] and increasing the release of immunoglobulin A (IgA) in the intestinal mucosa [127]. Although it is known that vitamin A has an impact on neutrophil differentiation and NK cell cytotoxicity, the exact mechanism remains elusive [128, 129].

### 3.6. The Effect of Vitamin A on *H. pylori* Infection

Beta carotene, the most important source of vitamin A production in the body [130], is an effective antioxidant inhibiting *H. pylori*-induced gastric inflammation, which not only reduces ROS but also interferes with the ROS-mediated inflammatory signaling such as MAPK and NF-*κ*B activation; consequently, *β*-carotene suppresses iNOS and COX-2 production. It also prevents neutrophil recruiting by reducing the expression of inflammatory mediators such as IL-8 [131]. Park et al. demonstrated that as a result of inhibiting ROS generation and NF-*κ*B activation, *β*-carotene reduces the *H. pylori*-induced production of TRAF1 and TRAF2 and cell proliferation in *H. pylori*-infected AGS cells [132]. A recent study investigated *H. pylori*-induced MMP expression and cell invasion and reported that *β*-carotene prevents MAPK-mediated MMP-10 expression and *H. pylori* invasion to AGS cell line through suppression of oxidative stress and upregulation of PPAR-*γ*-mediated catalase production [133]. Kim et al. reported that although exposure of AGS cells with low concentration (5 and 10 *μ*M) of *β*-carotene suppresses c-myc and cyclin E, a 10-fold higher concentration range (50 and 100 *μ*M) induces ROS production and apoptosis, decreases DNA repair protein expression, and activates caspase-3 [134].

Astaxanthin, the most efficient immune promoter among carotenoids, shifts the T helper cells regulation to Th2 as *H. pylori* changes the immunity balance between Th1 and Th2 toward Th1. Even though astaxanthin inhibits *H. pylori* growth and gastric inflammation, it does not decrease cytokine concentration in the infected tissue [135]. Furthermore, an in vitro study exhibited that astaxanthin can prevent *H. pylori*-induced apoptosis as well as its intracellular replication through regulating AGS cell line autophagy [136].

Although the role of provitamin A is not fully understood, several studies indicated a protective role for *β*-carotene in suppressing *H. pylori*-induced inflammation. On the other hand, the regulatory role of astaxanthin on the defect autophagy of infected cells highlights the importance of this carotenoid in preventing gastric adenocarcinoma.

### 3.7. Vitamin C

Albert Szent-Györgyi discovered vitamin C in the 1920s and found its relevance in the treatment and prevention of scurvy [137]. Owing to the nonfunctional gene encoding L-gluconolactone oxidase, this micronutrient classifies as an essential nutritional vitamin [138]. Vitamin C, a water-soluble antioxidant, is mainly present in its reduced form as ascorbic acid or oxidized form as dehydroascorbic acid and takes part in a variety of human bioactivity [139]. It is involved in the synthesis of collagen, noradrenaline, adrenaline, peptide hormones, and carnitine. Moreover, vitamin C contributes to gene transcription, regulation of translation, epigenetic mechanisms involved in the control of gene expression, elimination of ROS, and favoring iron absorption. However, excessive accumulation of this vitamin induces its pro-oxidant rather than antioxidant activity [140].

### 3.8. Vitamin C and Immune Function

In a dose-dependent manner, vitamin C promotes the proliferation of lymphocytes and increases antibody generation [141]. Vitamin C is involved in neutrophil and monocyte movements, neutrophil chemotaxis, NK cell activities, and lymphocyte delayed-type hypersensitivity (DTH) response [142]. Through contribution in apoptosis and clearance of the infection sites from spent neutrophils by macrophages, vitamin C prevents necrosis, neutrophil extracellular trap (NET) formation, and tissue damage [143]. High-dose supplementation of vitamin C is correlated with the increased relative abundance of the Lachnospiraceae bacterial family, which is predominant in the gut microbiota composition of healthy individuals and known to possess multiple anti-inflammatory and antioxidant effects [144].

### 3.9. The Effect of Vitamin C on *H. pylori* Infection

As an antioxidant, vitamin C can scavenge and eliminate ROS and reduce oxidative stress [145]. On the other hand, it is reported that vitamin C administration during acid-suppressive treatment can downregulate the overexpression of ROS, mucosal IL-8, and neutrophil infiltration in *H. pylori*-infected patients and possibly inhibit corpus gastritis [146]. It is long known that by inhibiting the generation of N-nitroso compounds that are strong carcinogens, vitamin C suppresses gastritis [147]. Another mechanism by which vitamin C can prevent gastric malignancy is that it increases apoptosis and G0/G1 cell cycle arrest in infected cells [148]. Vitamin C not only directly interferes with the energy metabolism or epigenome regulation of cancer stem cells but also stimulates collagen synthesis and indirectly suppresses tumor metastasis [149].

Although some studies decline the impact of *H. pylori* infection on vitamin C level [150], it has been recently indicated that infection with *H. pylori* impairs the secretion of vitamin C from serum to the stomach and thus decreases gastric juice vitamin C level [151]. According to Capurso et al., the observed reduction in gastric juice vitamin C levels in individuals with gastric atrophy is related to intragastric pH [152]. Intragastric pH level rises when hypochlorhydria develops, as is typical in gastric atrophy; thereby, ascorbic acid is transformed to the less active form of dehydroascorbic acid [153]. However, tissue vitamin C concentration is not affected by *H. pylori* infection and might still have a protective impact against gastritis [154]. It is noteworthy that CagA-positive strains have a significantly higher impact on vitamin C levels than CagA-negative strains [155]. On the other hand, vitamin C supplementation has a higher impact on CagA-positive strains [156]. Even though several studies examined the relation between vitamin C supplementation and *H. pylori* eradication and reported the restored gastric juice concentration of vitamin C after *H. pylori* eradication, the results of oral administration of vitamin C are still controversial [157].

Despite the contradictory reports regarding vitamin C interaction with *H. pylori*, this micronutrient plays a pivotal role in regulating the immune response and preventing tumor metastasis. However, high-dose administration of vitamin C might act as pro-oxidant rather than antioxidant and cause collateral damages.

### 3.10. Vitamin E

Vitamin E refers to a group of four tocopherols (*α*-, *β*-, *γ*-, and *δ*-tocopherols) as well as four tocotrienols (*α*-, *β*-, *γ*-, and *δ*-tocotrienols) found in food. These forms cannot be transformed into each other, and *α*-tocopherol meets the requirement of vitamin E in the human body [158]. The bioavailability of vitamin E is affected by dietary, absorption, metabolism, lifestyle, gender, and genetic polymorphisms [159]. It is noteworthy that LDL cholesterol is the principal plasma carrier of tocopherol. Consequently, vitamin E concentration is influenced by the number of plasma lipids and LDL cholesterol [160].

The bioactivity of vitamin E includes inhibiting oxidative stress, protecting cell membrane, regulating platelet aggregation, and stimulating the host immune defense [161]. Vitamin E promotes membrane repair by inhibiting lipid peroxidation, therefore interfering with phospholipid oxidized generation [162]. According to antioxidant activity, vitamin E also protects lipoproteins and membrane polyunsaturated fatty acids from oxidative damage [163]. By suppressing the function of protein kinase C, vitamin E interferes with platelet aggregation, nitric oxide generation in endothelial cells, superoxide anions expression in neutrophils and macrophages and regulates smooth muscle cell proliferation [164].

### 3.11. Vitamin E and Immune Function

Vitamin E stimulates the cytotoxic activity of NK cells, downregulates the expression of proinflammatory cytokines and PGE2, and thereby indirectly protects T cell function [165]. It has been reported that vitamin E modulates T cell functions including DTH response, the proliferation of lymphocytes, and IL-2 generation by directly promoting membrane integrity and signaling pathways in T cells [166]. Moreover, vitamin E downregulates IL-12 and IL-4 secretion from DCs and T cells, respectively. Furthermore, vitamin E can inhibit the NF-*κ*B pathway indirectly in DCs through the upregulation of Klotho, a membrane protein that mediates calcium transport into the cell [167].

### 3.12. The Effect of Vitamin E and C on *H. pylori* Eradication

An in vivo study investigated the effect of vitamin E supplementation on *H. pylori*-induced mucosal injury of male Mongolian gerbils and reported that the vitamin E-sufficient or supplemented group demonstrated no gastric ulceration. This study also reported that the enhanced expressions of CD11b/CD18 and keratinocyte-derived chemokine were downregulated by vitamin E administration, thereby inhibiting neutrophil infiltration [168]. Another study on Mongolian gerbils showed a short-term suppression of mucosal protein carbonyls and thiobarbituric acid reactive substances (TBARS) expression by vitamin E supplementation [169]. However, Zhang et al. reported no inhibitory activity for vitamin E on the colonization of *H. pylori* even at the high concentration (4096 mg/mL) using the agar dilution method [170]. This might indicate that vitamin E alone is incapable of suppressing *H. pylori* colonization but can come up with synergic effects in cosupplementation with antibiotic treatment.

The co-supplementation of vitamin E and C exhibits an additive activity because vitamin C can deoxide vitamin E after the oxidation of vitamin E during deoxidation of ROS, and accordingly, a few pieces of research indicated the benefit of antioxidant intake during *H. pylori* eradication (Table 2) [171–173]. On the contrary, several studies declined the beneficial effect of vitamin C and E administration on *H. pylori* eradication rate [174, 175]. Chuang et al. concluded that vitamin C and E supplementation not only has no inhibitory activity against *H. pylori* but also may reduce the efficiency of metronidazole-based therapy in patients infected with metronidazole-susceptible strains [176]. Interestingly, it has been reported that the mucosal content of *α*-tocopherol in the corpus of patients with *H. pylori* infection is lower than in the antrum or duodenum [177]. It is most likely due to the mobilization of antioxidant defenses to the regions of severe inflammation in the stomach [178]. Further large-scale population studies are required to determine the impact of vitamin E and C oral administration on *H. pylori* eradication rate. Moreover, in vitro investigations are needed to elucidate the mechanism of direct interaction between *H. pylori* and these vitamins.

### 3.13. Vitamin B

Vitamin B is composed of eight separate water-soluble important elements including B1 (thiamine), B2 (riboflavin), B3 (niacin), B5 (pantothenic acid), B6 (pyridoxine), B7 (biotin), B9 (folate), and B12 (cobalamin) [184]. Except for vitamin B3, that can be made of tryptophan, none of the B vitamins is produced by the human body and must be acquired from the diet or microbial sources such as gut microbiota [185]. However, the main sources for vitamin B12 are animal foods such as meat, fish, eggs, or dairy products [186].

### 3.14. Vitamin B and Immune Function

B vitamin family is involved in cellular metabolism pathways and immune regulation. Vitamin B6 mediates lymphocyte migration probably by regulating lipid mediator sphingosine 1-phosphate (S1P) which plays a pivotal role in cell trafficking; consequently, the deficiency of vitamin B6 is associated with lymphoid atrophy and lymphocyte reduction [187]. Vitamin B9 (folate) deficiency is associated with impaired cytotoxicity of NK cells and a substantial reduction in the number of T cells, NK cells, and most of all, B cells [188]. Folate-reduced state downregulates the expression of antiapoptotic Bcl2; therefore, the differentiated regulatory T (Treg) cells from naïve T cells will fail to survive [189]. An in vivo study exhibited that folate deficiency results in thymus and spleen atrophy, reduced proliferation of spleen lymphocytes, and decreased the number of circulating T lymphocytes; however, no effects were observed on the neutrophils [190]. The deficiency of vitamin B12 is related to a significant reduction in the number of lymphocytes, CD8^+^, and CD4^+^ as well as the activity of NK cells [191]. Vitamin B12 supplementation in Alzheimer's patients has demonstrated reduced expression of proinflammatory cytokines such as IL-8 and TNF-*α* and enhanced production of anti-inflammatory cytokine TGF-*β* [192].

### 3.15. Vitamin B and *H. pylori* Infection

One of the main complications of atrophic gastritis is vitamin B12 or cobalamin malabsorption [193]. The inefficacy of individuals to absorb food-bound or protein-bound cobalamin, while typically capable of absorbing free cobalamin, is defined as food-cobalamin malabsorption [194]. Shuval-Sudai and Granot exhibited a link between *H. pylori* infection and the frequency of reduced cobalamin concentration [195]. Acid suppressive therapy and *H. pylori*-induced changes in intragastric pH are most likely the main causes of vitamin B12 malabsorption [194]. Cobalamin malabsorption is common in the elderly due to achlorhydria and hypochlorhydria, bacterial overgrowth, and decreased intrinsic factor synthesis and secretion [193]. It has been proposed that *H. pylori* infection might have a significant role in the reduction of acid production and intrinsic factor secretion and hence develops vitamin B12 insufficiency [196]. Megaloblastic anemia is the typical symptom of vitamin B12 deficiency, yet it occurs only in 50% of vitamin B12-deficient individuals [197].

The link between folate and *H. pylori* infection has been studied only in a few pieces of research. Regarding adults, several researchers have shown an inverse connection between *H. pylori* infection and folate metabolism [13]. A reduction in folate absorption may occur as a result of reduced vitamin C levels in gastric juice and elevated intragastric pH, as is typical in *H. pylori* infection [198]. However, Ackam et al. reported no significant impact on folate concentration in *H. pylori*-infected children [199].

## 4. *H. pylori* and Minerals

Despite the presence of a micromolar amount of minerals in the human body, imperceptible alteration in their concentration may lead to significant modification of the host biology. The delicate interaction of minerals with the immune system as well as pathogenic bacteria highlights the importance of mineral homeostasis in preventing *H. pylori*-induced inflammation, as further discussed below (Table 3).

### 4.1. Iron

Despite the toxic properties of iron, it is a biologically vital element, contributing to hemoglobin production and heme and iron-sulfur cluster generation, and therefore regulates respiration, nucleic acid repair and replication, metabolic reactions, and immune response [224]. The regulation of iron concentration relies on hepcidin, ferroportin, and hypoxia-inducible factor-2 (HIF2). HIF2 regulates the production of proteins involved in iron absorption, while hepcidin, a liver-derived hormone, blocks ferroportin to inhibit iron absorption, storage, and recycling in duodenal enterocytes, hepatocytes, and reticuloendothelial macrophages, respectively [225]. In addition to general iron hemostasis, nutritional immunity as a dynamic mechanism allows the host to respond to pathogenic attacks and prevents bacterial growth by restricting bacterial access and intracellular availability to iron [226].

### 4.2. Iron and Immune Function

Oxygen burst by NADPH oxidase is a defensive response of neutrophils. By forming hydroxyl radicals in the interaction with hydrogen peroxide, iron is involved in the antimicrobial activity of neutrophils [200]. Polymorphonuclear leukocytes and monocytes release myeloperoxidase (MPO), an antimicrobial hemoprotein that requires the Fe^3+^/Fe^2+^ redox state for its activity [201]. Concerning T cells, iron changes the equilibrium between Th1 and Th2 cells towards Th2 and thereby increases the expression of IL-4 and inhibits the secretion of IFN-*γ* [202]. Iron supplementation, depending on the amount and form of iron, can trigger certain cellular events such as the polarization of macrophages and proinflammatory and anti-inflammatory cytokine secretion [207].

### 4.3. Iron and *H. pylori* Infection

Iron deficiency is a major marker of *H. pylori* infection, and thereby, multiple pieces of research attempted to elucidate this relationship. Although several studies indicated the correlation between *H. pylori* infection and iron deficiency or iron-dependent anemia, regardless of the presence or absence of peptic ulcer [228, 229], Savio et al. reported no relation regarding *H. pylori* infection in elder adults and unexplained iron insufficiency or anemia [230]. However, the origin and mechanisms underlying this possible connection are yet to be fully understood. It is suggested that owing to the higher iron requirement in *H. pylori* than other pathogens, competing for iron dietary leads to iron malabsorption in the host [231]. Moreover, the progression of *H. pylori*-induced infection can cause gastritis or duodenitis which might further result in gastrointestinal blood loss and anemia [203]. In addition, *H. pylori* induces hepcidin expression and thereby decreases iron secretion from macrophages [204]. The reduction in free iron elements can lead to cobalamin malabsorption due to the decreased iron-dependent cobalamin transporters [203].

### 4.4. Zinc

Zinc homeostasis mainly relies on intestinal absorption which occurs through zinc transporters on the apical and basolateral membrane of enterocytes and further by cellular zinc-binding protein metallothionein [232]. Calprotectin, a member of the S100A protein family, regulates the zinc concentration at the host-pathogen interface [233]. Insufficiency or overload of zinc level has an impact on growth, morphogenesis, immune response, neurosensory, and endocrine activity [234]. At the cellular level, zinc plays a pivotal role in proliferation, differentiation, and apoptosis [235].

### 4.5. Zinc and Immune Function

Zinc is an essential micronutrient to maintain the gut barrier by regeneration of intestinal epithelium, preserving the function and structure of membrane barrier and regulating the proliferation of immune cells [205]. The granulocyte recruitment, monocytes adhesion to epithelial cells, and ROS generation are dependent on zinc concentration. It has also been revealed that zinc deficiency is associated with a reduction in the number of granulocytes and NK cells and the phagocytic capacity of macrophages [206]. Low serum level of zinc increases the production of proinflammatory cytokines such as IL-1*β*, IL-6, and TNF-*α*, shifts the balance between Th1 and Th2 toward Th2, causes thymus atrophy, and reduces lymphopoiesis and antibody expression of T and B lymphocytes [207, 208].

### 4.6. Zinc and *H. pylori* Infection

*H. pylori* requires zinc to induce inflammatory activity including CagA translocation, Cag-T4SS pilus formation, NF-*κ*B activation, and consequently IL-8 expression [209]. To disrupt the activity of *H. pylori* virulence factors, the host antibacterial proteins including calprotectin (S100A8/A9 heterodimer) or calgranulin C (S100A12 homodimer) inhibit the access of pathogen to zinc elements [210]. Furthermore, Sempértegui et al. reported a negative correlation between *H. pylori*-induced gastric inflammation and gastric mucosa zinc concentration [236]. Interestingly, that innate immune defense exploits a high concentration of zinc and copper in the macrophage to eliminate the trapped bacteria. However, *H. pylori* evolved three zinc transport systems including CadA, CrdB-CzcAB complex, and CznABC system to survive high levels of zinc [210]. As this element plays a variety of roles in both *H. pylori* and the host immune system, further studies are required to elucidate the interplay between zinc bioavailability and *H. pylori*-induced inflammation. Moreover, the fate of trapped bacteria in the macrophage during the increased concentration of zinc needs to be further investigated.

### 4.7. Selenium

Selenium is a cofactor of glutathione peroxidase (GPx), which is a protective enzyme for cell membranes against oxidative damage [237]. The biologic effects of selenium are primarily mediated by the activity of selenoproteins that take part in a variety of cellular pathways, such as antioxidant defense to reduce oxidative stress and DNA damage, induction of phase II conjugating enzymes for carcinogen detoxification, and reduction of DNA adduct generation, prevention of cell proliferation, stimulation of DNA repair and apoptosis via the p53 tumor suppressor gene, and inactivation of proliferating cells [238].

### 4.8. Selenium and Immune Function

Selenium intake is involved in immune cell activation, differentiation, and proliferation. Specific selenoproteins also play a major part to preserve the equilibrium between reduced and oxidized molecules within the host cells [211]. It is apparent that selenium deficiency not only reduces the activation, maturation, and antimicrobial activity of T cells but also downregulates the expression of B cell-dependent antibodies. On the other hand, higher selenium intake skews the differentiation of CD4^+^ T cells toward Th1 by stimulating TCR signaling and consequently increases the production of proinflammatory cytokines [212, 213]. Selenium supplementation might decrease intra-abdominal adhesion formation and this is a possible mechanism by which selenium intake moderates inflammation [214].

### 4.9. Selenium and *H. pylori* Infection

Few studies that investigated the correlation between *H. pylori* and selenium serum concentration exhibited contradictory results. Camargo et al. reported that a high-risk population for gastric cancer demonstrated lower plasma selenium levels than low-risk areas [239]. However, Öztürk et al. demonstrated no substantial differences in serum selenium levels between *H. pylori*-infected and noninfected children [240]. On the contrary, it is suggested that selenium accumulates in gastric tissue during *H. pylori* infection owing to the excessive ROS production [241].

### 4.10. Copper

Copper is a vital nutritional element of human physiology, which is required as a catalytic cofactor in several enzymatic processes, such as an allosteric enzyme component and an antioxidant which plays a key role in the oxidant defense system [242]. In the adult human body, the copper content is predicted to be between 50 and 120 mg. It is presented in large concentrations in the liver and brain, and to a lesser extent in the kidneys, heart, and pancreas. Microcytic anemia, leukopenia, osteoporosis, new subperiosteal bone growth, and epiphysis fibrosis can all be developed from copper deficiency [243].

### 4.11. Copper, Immunity, and *H. pylori* Infection

Copper is involved in leukocyte proliferation and maturation; thereby, its deficiency decreases the number of neutrophils and impairs the activity of macrophages, neutrophils, and monocytes [215]. In vivo studies have demonstrated that copper diet levels impact the susceptibility of the host to pathogenic infection, and thereby sensitize the animal models to infection, extend the infection duration, and increase the mortality rate [244].

According to Janjetic et al., serum copper levels in children might be linked to gastric *H. pylori* infection; however, further studies are required to confirm this correlation [245]. Regarding adults, studies indicated no significant difference in serum copper levels between *H. pylori*-infected and noninfected individuals. Furthermore, no substantial difference in serum copper concentration is detected concerning the successful eradication of *H. pylori*. Wu et al. reported that although triple therapy significantly reduces the selenium serum levels, copper levels remained the same after *H. pylori* eradication [216].

### 4.12. Magnesium

Magnesium (Mg^2+^) is a cofactor of numerous enzymes engaged in critical metabolic processes required for bacterial survival. Pathogenic bacteria express specific Mg^2+^ absorption mechanisms to circumvent the Mg^2+^ restriction within the human host [246]. On the other hand, in the human body, Mg^2+^ is mainly absorbed in the small intestine through paracellular transport and membrane transporters. Due to the poor expression of claudins in the small intestine, paracellular transport is the most common mechanism for magnesium transportation [247].

### 4.13. Magnesium, Immunity, and *H. pylori* Infection

It is long known that the antioxidant and anti-inflammatory activities of magnesium support the immune system against several diseases [248]. Mg^2+^ decreases macrophage activation, downregulates cytokine expression in activated macrophages at the mRNA level, and impedes the NF-*κ*B activation [217]. Low serum levels of magnesium increase apoptosis, oxidative stress, and proinflammatory cytokine production [218]. Magnesium deficiency also decreases the number of CD8^+^ T cells and possibly leads to reduced IFN-*γ* concentration [219].

In *H. pylori*, phosphate metabolism, particularly the catabolism of phosphonates, is strongly reliant on Mg^2+^, which can serve as a significant supply of phosphorous. *H. pylori* may degrade the phosphonate and phenyl phosphonate and use them as a single supply of phosphate. This catabolism was demonstrated to be increased by exogenous MgCl_2_ and inhibited by the ethylenediaminetetraacetic acid (EDTA) [220].

### 4.14. Nickel and Immune Function

There are contradictory results regarding the immunomodulation capacity of nickel, yet solid evidence indicates that nickel potentially suppresses the host immune response [221]. The exposure of immune cells to a low concentration of nickel can influence the normal activity of the immune system [249]. Splenic NK cells were demonstrated to express IFN-*γ* as a direct or indirect response to nickel detection in the cell culture system. Substantial reduction in the presence of nickel-reactive cells following the depletion of NK cells in an animal model was further reported to confirm the in vitro results [222]. The involvement of CD25^+^ Treg in the activation of nickel-specific T cells is suggested to promote nickel tolerance and potentially prevent the development of allergic contact dermatitis [250].

### 4.15. Nickel and *H. pylori* Infection

In addition to zinc and iron, *H. pylori* requires the nickel transition to survive in the host and promotes its pathogenic activity. By leveraging nickel-containing metalloenzymes such as Ni-Fe-hydrogenase and urease, *H. pylori* can survive the acidic condition of the human stomach [223]. Urease, as one of the most prevalent enzymes in the *H. pylori* proteome, consists of 24 nickel atoms to generate ammonia and bicarbonate from urea [251]. However, excessive concentration of nickel in the bacterial cell causes mismetallation of cellular enzymes, which can further impair its physiological function [252]. *H. pylori* is likely to come into contact with micromolar amounts of transition metals such as nickel from the host food, which can alter nickel homeostasis as well as the activity of hydrogenase and urease [253]. As a result, *H. pylori* can extremely control both the nickel import and export functions in its cellular nickel economy [254]. Nickel is transported into the bacterial cell by a NixA permease [255] and the FrpB4 outer membrane protein in a TonB-dependent manner [256]. A recent study demonstrated that SlyD, a protein combining peptidyl-prolyl isomerase (PPIase), chaperone, and metal-binding properties, acts as the gatekeeper of nickel import by regulating the function of Niu permease [257]. The promiscuous CznABC transporter facilitates nickel efflux and thereby enhances nickel resistance in *H. pylori* [253]. Campanale et al. exhibited that keeping patients on a nickel-free diet improves *H. pylori* treatment, suggesting the importance of nickel in *H. pylori* pathogenesis [223].

## 5. Concluding Remarks

In this review, we discussed the effects of vitamins A, D, C, E, B6, B9, and B12, as well as the key minerals such as zinc, iron, copper, nickel, and selenium on immune function and their involvement in *H. pylori* infection and eradication. The antioxidant activity of vitamins can reduce oxidative stress and further suppress *H. pylori*-induced inflammation. Moreover, in vitro studies demonstrated promising anti-*H. pylori* mechanisms for vitamin administration in a dose-dependent manner. On the other hand, several researchers reported an inverse relation between *H. pylori* infection score and vitamin concentration in the gastric juice. Furthermore, only a few studies demonstrated a negative impact of vitamin administration on *H. pylori* treatment, while other papers exhibited a substantial or comparable advantage. However, further studies are needed to investigate a large-scale population of infected individuals and elucidate the impact of vitamin supplementation on *H. pylori* eradication rate and side effects.

On the contrary, certain minerals are involved in *H. pylori* pathogenesis as well as the host immune response. The delicate interplay between minerals, immune system, and *H. pylori* infection remains to be fully elucidated. The contradictory reports regarding alteration in the host serum levels of minerals after *H. pylori* treatment require further examination. Additionally, in vitro studies are of great significance to clarify the interaction between mineral concentrations, *H. pylori* pathogenesis, and the host cellular pathways.

## Figures and Tables

**Table 1:** The effects of vitamin D deficiency on *H. pylori* eradication.

Country	Study design	Eradication regimen	Antioxidant	Confirming *H. pylori* infection test	Confirming *H. pylori* eradication		Eradication rate			Antioxidant level (ng/mL)			References
					Method	Time (weeks after therapy)	Successful eradication	Failed eradication	*P* value	Successful eradication	Failed eradication	*P* value	
Egypt	Randomized controlled trial	Amoxicillin 1000 mg bid, clarithromycin 500 mg bid, esomeprazole 20 mg bid, 14 days	25 (OH) D	ME-NBI, SAT	SAT	4	105/150	45/150	NA	27.41 ± 7.1	14.7 ± 4.5	*P* < 0.001	[108]
China	Randomized controlled trial	Amoxicillin 1000 mg bid, clarithromycin 500 mg bid, colloidal bismuth tartrate capsule 220 mg bid, esomeprazole 40 mg bid or rabeprazole 20 mg bid, 14 days	25 (OH) D	UBT	UBT	4-8	355/415	60/415	NA	322/355 ≥ 10 (ng/mL), 33/355< 10 (ng/mL)	13/60 ≥ 10 (ng/mL), 47/60< 10 (ng/mL)	*P* < 0.005	[109]
China	Randomized controlled trial	Amoxicillin 1000 mg bid, clarithromycin 500 mg bid, bismuth potassium citrate 220 mg bid, esomeprazole 20 mg bid, 14 days	25 (OH) D	UBT	UBT	4	124/160	36/160	P = 0.677	19.87 ± 6.35	15.09 ± 7.72	*P* < 0.05	[110]
Israel	Randomized controlled trial	Based on the individual conditions	25 (OH) D	UBT, SAT	UBT, SAT	0	45,821/61,921	16,100/61,921	NA	19.34 ± 9.55	18.64 ± 9.61	*P* < 0.001	[105]

qd, once a day; bid, twice a day; tid, three times a day; qid, four times a day; UBT, urea breath test; SAT, stool antigen test; ME-NBI, magnifying endoscopy with narrow band imaging; NA, not available.

**Table 2:** The effects of vitamin supplementation on *H. pylori* eradication.

Country	Study design	Eradication regimen	Antioxidant supplementation	Confirming *H. pylori* infection test	Confirming *H. pylori* eradication		ITT eradication		PP eradication		References
					Method	Time (weeks after therapy)	Without antioxidant	With antioxidant	Without antioxidant	With antioxidant	
Taiwan	Randomized controlled trial	Lansoprazole 30 mg bid, amoxicillin 1000 mg bid, metronidazole 500 mg bid, 7 days	(vitamin C 250 mg + vitamin E 200 mg) bid, 7 days and (vitamin C 250 mg + vitamin E 200 mg) qd, 42 days	H, C	H, C, UBT	8	29/49	22/55	29/45	22/50	[176]
UK	Randomized controlled trial	Metronidazole 400 mg tid, bismuth chelate 120 mg qid, tetracycline 500 mg qid, 14 days	(vitamin C 200 mg + vitamin E 50 mg) bid, 28 days	H, C, RUT	H, C, RUT	4	17/30	19/29	17/25	19/24	[179]
Iran	Randomized controlled trial	Amoxicillin 1000 mg bid, omeprazole 20 mg bid, clarithromycin (250 mg in antioxidants group and 500 mg in without antioxidants group) bid, 14 days	Vitamin C 250 mg bid, 14 days	H, RUT	UBT	4	89/100	99/114	89/100	99/114	[180]
Turkey	Randomized controlled trial	Amoxicillin 1000 mg bid, clarithromycin 500 mg bid, bismuth subcitrate 300 mg qid, lansoprazole 30 mg bid, 14 days	(vitamin C 500 mg + vitamin E 200 mg) bid, 30 days	H, RUT	UBT, SAT	4	48/80	73/80	48/75	73/78	[172]
Iran	Randomized controlled trial	Amoxicillin 500 mg bid, metronidazole 500 mg bid, bismuth 240 mg bid, omeprazole 20 mg bid, 14 days	Vitamin C 500 mg qd, 14 days	RUT	UBT	4	68/140	110/141	79/140	117/150	[181]
Turkey	Randomized controlled trial	Amoxicillin 1000 mg bid, clarithromycin 500 mg bid, lansoprazole 30 mg bid, 14 days	(vitamin C 500 mg + vitamin E 200 mg) bid, 30 days	H, RUT	UBT	4	17/40	51/80	17/38	51/77	[182]
Turkey	Randomized controlled trial	Amoxicillin 1000 mg bid, Clarithromycin 500 mg bid, Lansoprazole 30 mg bid, 14 days	(vitamin C 500 mg + vitamin E 200 mg) bid, 30 days	H, RUT	UBT	4-6	18/40	132/160	18/38	132/157	[171]
Egypt	Randomized controlled trial	Amoxicillin 1000 mg bid, clarithromycin 500 mg bid, esomeprazole 20 mg bid, 14 days	Vitamin C 500 mg bid, 28 days	ME-NBI, H, SAT	SAT	4	31/50	34/50	31/44	34/46	[183]

qd, once a day; bid, twice a day; tid, three times a day; qid, four times a day; H, histopathologic examination; UBT, urea breath test; RUT, rapid urease test; SAT, stool antigen test; C, culture; ME-NBI, magnifying endoscopy with narrow band imaging; ITT, intention-to-treat; PP, per protocol.

**Table 3:** The influence of minerals bioavailability on the host and *H. pylori* pathogenicity.

Mineral	Functions	Deficiency	Interaction with *H. pylori*
Iron	Involved in anti-microbial activity of neutrophils, polymorphonuclear leukocytes and monocytes [200, 201]; Changes the equilibrium between Th1 and Th2 cells towards Th2 [202]	Cobalamin malabsorption [203]	*H. Pylori*-induced gastric bleeding leads to anemia [203]; *H. Pylori*-induced hepcidin expression reduces iron secretion from macrophages [204]
Zinc	Maintains gut barrier [205]; Involved in granulocyte recruitment [125]; Involved in monocytes adhesion to epithelial cells [125]; Involved in ROS generation [125]	Reduces the number of granulocytes and NK cells [206]; Reduces macrophages activity [206]; Increases pro-inflammatory cytokines production [207]; Shifts the balance between Th1 and Th2 toward Th2 [207, 208]; Reduces lymphopoiesis and antibody expression of T and B lymphocytes [207, 208]	Required for *cag*PAI functionality [209]; Calprotectin or calgranulin C restricts *H. pylori* access to zinc [210]; High concentrations of zinc eliminate bacteria [210]
Selenium	Preserves the equilibrium between reduced and oxidized molecules within cells [211]; Skews the differentiation of CD4+ T cells toward Th1 [212, 213]; Decreases intra-abdominal adhesion formation [214]	Reduces the activation, maturation, and anti-microbial activity of T cells [212, 213]; Reduces the expression of B cell-dependent antibodies [212, 213]	Contradictory results
Copper	Involved in leukocyte proliferation and maturation [215]	Impairs the activity of macrophages, neutrophils, and monocytes [215]; Decreases the number of neutrophils [215]	No correlation with *H. pylori* eradication [216]
Magnesium	Decreases macrophage activation [217]; Downregulates cytokines expression in activated macrophages [217]; Impedes the activation of NF-*κ*B [217]	Increases apoptosis, oxidative stress, and production of pro-inflammatory cytokines [218]; Decreases the number of CD8+ T cells [219]	Involved in phosphonate and phenyl phosphonate degradation in *H. pylori* [220]
Nickel	Potentially suppresses immune response [221]; Increases IFN-*γ* secretion from NK cells [222]	Nickel-free diet may improve *H. pylori* eradication [223]	Required for Ni-Fe-hydrogenase and urease [223]
